# 

**Published:** 1901-09

**Authors:** 


***NEW PREMIUM.—To each new, cash subscriber, an Improved Gauze Packer Free.***

***NEW PREMIUM.—To each new, cash subscriber, an Improved Gauze Packer Free.***

